# Aspect of Thrombolytic Therapy: A Review

**DOI:** 10.1155/2014/586510

**Published:** 2014-12-10

**Authors:** Md. Ramjan Ali, Mohammad Salim Hossain, Md. Ariful Islam, Md. Saiful Islam Arman, Golam Sarwar Raju, Prianka Dasgupta, Tasnim Fariha Noshin

**Affiliations:** ^1^Department of Pharmacy, Noakhali Science and Technology University, Sonapur, Noakhali 3814, Bangladesh; ^2^Department of Pharmacy, University of Rajshahi, Rajshahi 6205, Bangladesh

## Abstract

Thrombolytic therapy, also known as clot busting drug, is a breakthrough treatment which has saved untold lives. It has been used in the clinical area to treat venous and arterial thromboembolic complaints which are a foremost cause of death. In 1761, Morgagni lead the way of thrombolytic therapy. Now day's different types of thrombolytic drugs are currently available in market: alteplase, anistreplase, urokinase, streptokinase, tenecteplase, and so forth. Thrombolytic therapy should be given with maintaining proper care in order to minimize the risk of clinically important bleeding as well as enhance the chances of successfully thrombolysis of clot. These cares include preinfusion care, during the infusion care, and postinfusion care. Besides proper knowledge of contraindication, evolutionary factor, and combination of drug is essential for successful thrombolytic therapy. In these review we discussed about these aspect of thrombolytic therapy.

## 1. Introduction

A blood clot (thrombus) develops in the circulatory system which consolidates a mechanism in human body to repair the injured blood vessel [1]. If thrombus is formed when it is not needed, this can produce significant consequences [2] like embolism, ischemia, heart attack, stroke, and so forth [3]. Embolism occurs when blood clot is formed inside a blood vessel or an artery and remains there which fully or partially block blood supply to a part of body resulting potentially severe consequences. For example, a pulmonary embolism leads inexplicable breathing difficulty, hemoptysis, and chest pain when one or more arteries in lung are blocked by embolus [4]. Blood clot can block blood flow or oxygen to tissue which results in ischemia. Cardiac ischemia appears when blood flow to cardiac muscle becomes fully or partially restricted resulting shortness of breath, syncope, angina, myocardial infarction, cardiac arrhythmia, or even death [5]. Blood clots may also disrupt the flow of blood to the brain, leading to an ischemic stroke [6]. An ischemic stroke can occur as a result of a barrier within a blood vessel supplying blood to the brain (thromblic stroke) or embolus produced from clot somewhere else in the body and travels to block a small artery in the brain (embolic stroke). Sometimes blood clot forms in the heart and get trapped in the brain's narrow arteries (cerebral stroke). These consequences deprive the brain of necessary oxygen which result in permanent brain cell death in and around the affected area [7].

## 2. Thrombolytic Therapy: Clarification

Thrombolytic therapy is a treatment to get rid of problems raised due to blood clot or thrombus to renovate function to the affected area [8]. Thrombolytic agent, which is also known as clot buster, has saved untold lives. Thrombolytics afford longer-term benefits for survivors, who have just a 5% mortality rate at one year [9]. Thrombolytic agent is commonly used for

1. venous thrombosis,
2. pulmonary embolism,
3. myocardial infarction,
4. arterial thromboembolism,
5. acute ischemic stroke [10].

### 2.1. Classification of Thrombolytic Agent

Thrombolytic agents can be classified according to their generation as shown in Table 1 [11].

### 2.2. Basic Pharmacology of Thrombolytic Drug

**Figure 1:**
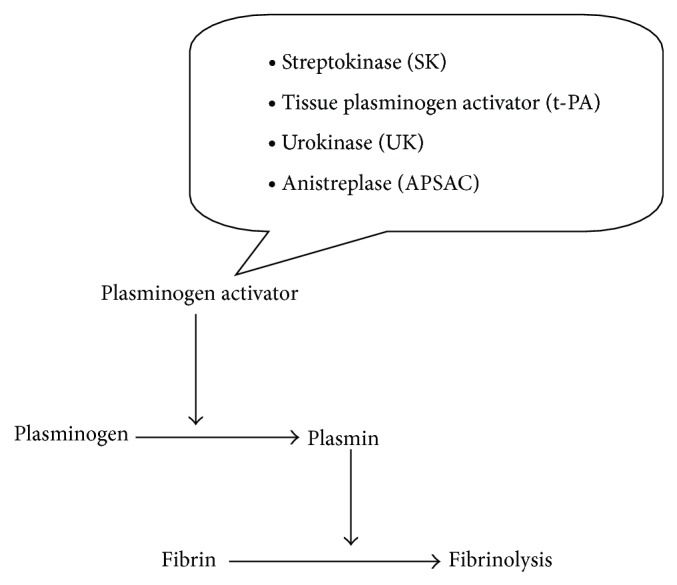
Schematic representation of fibrinolysis.

Thrombolytic drugs rapidly lyse thrombi by catalyzing the formation of the serine protease plasmin. Schematic representation of fibrinolysis is shown in Figure 1. Several types of thrombolytic drugs are commonly used worldwide. Their pharmacology are summarized below.

#### 2.2.1. Tissue Plasminoogen Activator

Tissue plasminoogen activator is a serine protease consisting of a single chain of 527 amino acids. Its molecular weight is about 70,000 daltons [12, 13]. tPA binds to fibrin on the surface of the clot which activates fibrin-bound plasminogen. Plasmin is cleaved from the plasminogen associated with the fibrin. Fibrin molecules are broken apart by the plasmin and the clot dissolves [14]. Normally, circulating *α*_2_-antiplasmin inactivates plasmin, but therapeutic doses of tPA (and SK) lead to sufficient plasmin formation to overwhelm the limited circulating concentrations *α*_2_-antiplasmin. Human t-PA is manufactured as altplase by means of recombinant DNA technology [15].

Reteplase is another recombinant t-PA from which several amino acid sequences have been removed. Due to lack of fibrin binding domain and less fibrin specificity, reteplase is cheaper to produce than t-PA. Tenecteplase (TNK-tPA) is a mutant form of t-PA that has a longer half-life. Tenecteplase is slightly more fibrin-specific than t-PA [16].

#### 2.2.2. Streptokinase

Streptokinase is a protein (but not an enzyme in itself) produced by various strains of h-hemolytic streptococci having a molar mass of 47 kDa and is made up of 414 amino acid residues. The protein exhibits its maximum activity at a pH of approximately 7.5 and its isoelectric pH is 4.7 [15]. This protein is single chain polypeptide that combines with the proactivator plasminogen. This enzymatic complex prompts the alteration of inactive plasminogen to active plasmin and, thus, exhibits fibrinolytic activity.

#### 2.2.3. Urokinase

Urokinase is a human enzyme synthesized by the kidney that directly converts plasminogen to active plasmin [12]. Urokinase has high molecular weight of 5400 Daltons. It consists of three domains: the serine protease, the kringle domain, and the growth factor domain with 411 residue protein [17, 18]. Naturally occurring inhibitors in plasmin hinder plasmin to work itself. However, the absence of inhibitors for urokinase and the streptokinase-proactivator complex permits their use clinically. Plasmin formed inside a thrombus by these activators is protected from plasma antiplasmins, which allows it to lyse the thrombus from within.

#### 2.2.4. Anistreplase

Anistreplase (anisoylated plasminogen streptokinase activator complex; APSAC) consists of a complex of purified human plasminogen and bacterial streptokinase that has been acylated to protect the enzyme's active site. When administered, the acyl group spontaneously hydrolyzes, freeing the activated streptokinase-proactivator complex. This product (recently discontinued in the USA) allows for rapid intravenous injection, greater clot selectivity (i.e., more activity on plasminogen associated with clots than on free plasminogen in the blood), and more thrombolytic activity [16].

### 2.3. Epigrammatic Record of Thrombolytic Drug

Perhaps the field of fibrinolysis originated with Morgagni in 1761 [19]. He observed that blood was not clotted after sudden death. Denis in 1838 [20] observed spontaneous clot dissolution followed by Denys and De Marbaix in 1889 [21] postulated a dormant blood fibrinolytic enzyme, but the occurrence of post-mortem fibrinolysis was oppressed by Skundina et al. [22]. Finally, Yudin [23] in the mid-1930s provided a source of unclotted blood for transfusion.

The streptokinase era dates back to 1933, while Tillett and Garner [24] discovered the agent through sheer serendipity, who called it fibrolysin. But first test was carried out on human in 1947 to lyse chronic thoracic empyemas with considerable success. Due to difficulties in purifying the protein the intravenous administration of streptokinase was delayed. In the 1960s, Behringwerke AG and Kabi Pharmacia made the drug accessible for prevalent therapeutic use. A significant success came during first trial using streptokinase with acute myocardial infarction, published between 1978 and 1988, compared with conservative treatment or placebo [25–27].

In 1980s, there has been an explosion of works in thrombolytics therapy where melanoma tPA was first demonstrated in rabbits with experimental pulmonary embolus in vivo. Tissue plasminogen activator (tPA) originally developed in the mid 1981s for acute coronary artery occlusion [28, 29]. Recombinant tPA (rtPA) was produced in late 1981s after molecular cloning techniques were used to express human tPA DNA. A predominantly single-chain form of rtPA was eventually accepted in the US for the treatment of acute MI and massive pulmonary embolism [30]. A recent study provides the evidence to use rtPA in the treatment of acute ischemic stroke [31]. An effort was taken later to lengthen the duration of tPA. Human gene for tPA was modified by genetic engineering where different amino acids occur at three locations to yield tencepteplase (TNK-tPA). This modification gives TNK-tPA a longer half-life and allowed successful administration as a single bolus in contradiction of the infusion needed for rtPA. TNK-tPA possesses relative resistance to plasminogen inhibitor and more fibrin specific than either tPA [32, 33]. Recent investigation has found TNK-tPA to be useful in embolic stroke [34].

The fibrinolytic potential of human urine was first described by Macfarlane and Pilling in 1947 [35]. The active molecule was extracted, isolated, and named “urokinase” (UK) in 1952 [36]. A precursor of UK was discovered in urine in 1979 [37]. Prourokinase was characterized and subsequently produced by recombinant technology using* Escherichia coli* (nonglycosylated) or mammalian cells (fully glycosylated). This single-chain form is a dormant zymogen, inert in plasma but stimulated by kallikrein or plasmin to form potent 2-chain UK, which accounts for amplification of the fibrinolytic progression. As plasmin is formed, more prourokinase is turned into active urokinase, and the process is continual. Given the possible favor of prourokinase over urokinase, Abbott Laboratories generated a recombinant form of prourokinase (r-proUK) from a murine hybridoma cell line. Named Prolyse, this recombinant agent is turned into active 2-chain UK by plasmin and kallikrein. Prolyse has been conscious in the settings of MI, stroke, and peripheral arterial occlusion. McNamara and Fischer [38] were the first to tell the use of urokinase for regional thrombolytic treatment, employing a high-dose protocol featuring graded, stepwise reductions in dose as the infusion development. For the first time, clinicians realize comfortable with the risk-benefit equation when giving patients with thrombolytic agents.

### 2.4. How Are Thrombolytics Administered?

Thrombolytic drugs are administered intravenously and are necessary to give as soon as possible after the patient progresses the signs and symptoms of STEMI; the earlier they are given the better will be noticed. The American College of Cardiology and the American Heart Association suggest that in order for the thrombolytic drugs to be most successful, they should be administered in 30 minutes of the patient's entrance at the hospital [39].

#### 2.4.1. Nursing Care of Patient during Receiving Thrombolytic Therapy

The steps included during nursing care are shown in Table 2 [40].

#### 2.4.2. Contraindications for Using Thrombolytic

Thrombolytics are predominantly safe; complications related with bleeding are the major problem. Study revealed that 11% of all patients who receive thrombolytics have reasonable bleeding. Among them 0.3%–1.3% experience intracranial hemorrhage. Contraindications for using thrombolytic can be classified into two ways—absolute contraindications and relative contraindications.

Absolute contraindications are

- vascular lesions,
- severe, uncontrolled hypertension,
- recent cranial surgery or trauma,
- brain tumor,
- ischemic stroke in two to three months,
- active bleeding (except for normal menstrual bleeding).

Relative contraindications can be included:

- ischemic stroke > three months prior,
- active peptic ulcer,
- current use of anticoagulant drugs,
- pregnancy,
- prolonged/traumatic CPR ≤ three weeks prior,
- major surgery ≤ three weeks prior,
- internal bleeding in two to four weeks [39, 41, 42].

### 2.5. Factors Related with Delay in Thrombolytic Therapy

There is indomitable evidence that exact treatment such as thrombolytic develops the chances of an amicable outcome when administered within a suitable time-window [43–45]. It is predictable that the effect of thrombolytic therapy decreases swiftly over time even within the 3-hour window, consequential in the concept of “time is brain” [46]. Despite the evidence, only a few are prehospital and intrahospital delay. Prehospital delay includes the two key factors of decision and transportation periods, and decision time implies the break between the onsets of symptoms until the patient observes the gravity of the problem and seeks medical assistance. Decision delay is more prominent than are the other factors of the patients' treatment (transfer and intrahospital delays). Intrahospital delay refers to delay of thrombolytic therapy due to hospital's administration systems like delay in registration and billing issues or because of untrained staff [47, 48].

### 2.6. Education, Training, and Equipment for Effective Thrombolytic Therapy

The effectiveness and safety of prehospital thrombolysis is relying on several prerequisites [49–53]:

- Prehospital personnel should be trained to identify symptoms and management of STEMI and its earlier complexities (pain, bradycardic arrhythmias, and ventricular fibrillation/ventricular tachycardia).
- Diagnoses of STEMI by a 12 lead ECG with or without computer help for diagnosis and/or data transmission.
- Intravenous access to be conventional and the administration of reperfusion therapy to be pioneered within a treatment procedure/clinical principle and affirmed by a thrombolysis checklist.
- During transportation rhythm observation, availability of a defibrillator and modern cardiac life support are required.
- Precaution receiving hospital of imminent arrival of the patient supported by (if available) electronic transmission of the 12 lead ECG.
- On-going quality promise.

#### 2.6.1. Patient Education

Patient's education plays vital role for proper implementation of thrombolytic therapy which are followed [54].

- Instruct patient about procedures and their necessity prior to beginning thrombolytic therapy.
- Instruct patient that frequent vital signs must be taken.
- Instruct patient that activity will be limited during infusion and that pressure dressing may be needed to prevent any active bleeding.
- Advise patient about assessments and why they are necessary.
- Advise patient that cardiac rhythm will be observed during therapy.
- Instruct patient about increased risk for bleeding, activity restriction, and frequent monitoring during this time.

## 3. Adjunctive Drug Treatment

### 3.1. Oxygen

Oxygen is commonly administered during the management of patients during thrombolysis. Its routine use in patient with pulse oximetry above 95% has been questioned primarily due to the possibility of vasoconstriction and limited evidence of benefit [55]. Oxygen remains an important adjunctive therapy in the presence of left ventricular failure although it is better provided as part of continuous positive airway pressure. Oxygen should routinely be administered in the presence of arrhythmias (e.g., ventricular tachycardia) [56], hypotension/hypoperfusion and any postcardiac arrest.

### 3.2. Aspirin

All patients with suspected acute coronary syndrome (unstable angina, non-STEMI, and acute STEMI) should be considered for prehospital aspirin treatment. It blocks prostaglandin formation which in turn leads to a decrease in the synthesis of thromboxane A2. Thromboxane A2 results platelets to adhere to each other. Despite this, aspirin is often withheld either due to concerns over allergy, adverse drug interactions (e.g., Warfarin), confusion due to chronic ongoing use of aspirin, or uncertainty of diagnosis [27, 57].

### 3.3. Clopidogrel

Clopidogrel is a strong platelet inhibitor and the antiplatelet benefits of clopidogrel in combination with aspirin are to lessen ischaemic events in non ST-elevation acute coronary syndromes (PCI) [58] and in patients undergoing percutaneous coronary intervention.

### 3.4. Newer Oral Antiplatelet

New developed oral antiplatelets such Prasugrel and Ticagrelor are now existing with potentially developed antiplatelet as Ticagrelor produces a more profound and reliable antiplatelet effect than that of clopidogrel [59] but as yet to be studied in conjunction with thrombolytic therapy.

### 3.5. Heparin

Heparin is considered to be effectual and is regularly given as an adjunct for PCI and thrombolytic therapy although it is frequently withheld in the first 24 hours in those patients receiving Streptokinase. The role of heparin is mainly to decrease reinfarction but the combination of dual antiplatelet therapy, thrombolysis, and heparin may amplify the risk of bleeding. The American Heart Association strategies call for careful weight based dosing of heparin with thrombolytic therapy in STEMI [60].

### 3.6. Steroids and Antihistamines

The regular administration of steroids and antihistamines to prevent hypotension/bradycardia specially in association with Streptokinase complicates the administration procedures and is improbable to avert hypotension as the cause of Streptokinase induced hypotension is principally due to speed of administration [61, 62] and the exploit of bradykinin activated by Streptokinase [63].

### 3.7. Some Selected Pharmacokinetic and Pharmacodynamics Parameters of Thrombolytic Agents

Some selected pharmacokinetic and pharmacodynamics parameters of thrombolytic agents are given in Table 3 [10, 11, 16, 64].

## 4. Recent Clinical Trials

Sezer and his colleagues studied to investigate the reflections of the progress in microvascular perfusion provided by adjuvant intracoronary streptokinase (ICSK) on late-phase infarct size and left ventricular volumes and functions. In this study, it had been demonstrated that low-dose ICSK given immediately after primary percutaneous coronary intervention significantly limits long-term infarct size and preserves left ventricular volumes and functions [65].

Gupta and his colleagues hypothesized throughout their clinical trial that streptokinase can be a useful adjunct in expanding nonoperative management of infected walled off pancreatic necrosis while not responding to pigtail catheter drainage and saline irrigation [66].

Sugimoto and his colleagues studied 93 patients and finally revealed that low-dose t-PA combined with subtherapeutic heparin is equally efficacious and safe compared with urokinase. Infusions with t-PA were significantly shorter and less expensive than those with urokinase [67].

Roth conducted a double-blind, multicenter, parallel-group trial for four years included patients from 18 to 80 years. He provided sufficient data that recombinant tissue plasminogen activator is useful in treatment of acute ischemic stroke [68].

Wang and his colleagues compared urokinase 2 h and urokinase 12 h regimen in treating acute pulmonary embolism in a randomized, controlled, multicenter trial. This study demonstrated that urokinase h (20 000 IU/Kg) regimen displayed similar efficacy and safety as the urokinase 12 h regimen in treating acute pulmonary embolism with either hemodynamic instability or massive pulmonary artery obstruction. Given the convenience, lower cost and the similar efficacy and safety as the urokinase 12 h regime, they suggested that body weight adjusted urokinase 2 h regimen could be used for pulmonary embolism treatment [69].

Wang and his colleagues hypothesized that among patients with transient ischemic attack or minor stroke that can be treated within 24 hours after the onset of symptoms. He with his colleagues observed that combination of clopidogrel and aspirin is superior to aspirin alone for reducing the risk of stroke in the first 90 days and does not increase the risk of hemorrhage [70].

Morrow and his colleagues revealed that prehospital administration of reteplase is a feasible approach to accelerating reperfusion in patients with STEMI. Valuable time savings can be achieved in the setting of contemporary transport and door-to-drug times and may translate into an improvement in clinical outcomes [71].

Oldgren and his colleagues demonstrate that in patients with a recent acute coronary syndrome, the addition of a new oral anticoagulant to antiplatelet therapy leads to a modest reduction in cardiovascular events but a substantial increase in bleeding. These results are most pronounced when oral anticoagulants are combined with dual antiplatelet therapy with aspirin and clopidogrel [72].

## 5. Concluding Remarks

Understanding of thrombolytic therapy is needed for development in pharmacological reperfusion. Chronicle history of thrombolytic therapy was recorded since 1761 and till now it saved untold lives. But it is conspicuous that failure of thrombolytic therapy due to some barrier. It is not inexplicable to overcome this barrier. Proper decision making, trained personnel and equipment, and patients education could be impetus for effective thrombolytic therapy.

## Figures and Tables

**Table 1:** 

Generation of thrombolytic drug	Fibrin specific	Nonfibrin specific
First	*⋯*	Urokinase^*^
	*⋯*	Streptokinase^*^

Second	Recombinant tissue plasminogen activator^*^ (t-PA)	Prourokinase (scum-PA)
	Alteplase	Sk-plasminogen activating complex^*^ (APSAC)

Third	Tenecteplase^*^ (TNK-tPA)	*⋯*
	Reteplase^*^	*⋯*
	Monteplase	*⋯*
	Lanoteplase	*⋯*
	Pamiteplase	*⋯*

^*^Approved for clinical use.

**Table 2:** 

Preinfusion care	During the infusion	Postinfusion care
(a) Obtain a whole health history together with recent surgeries or trauma, allergies, drug history, and possible drug interactions. (b) Assess for contraindications to thrombolytic therapy. (c) Assess lab values: aPTT, hemoglobin (Hgb) PT, and platelet count hematocrit (Hct).	(a) Assess and record very important signs and the infusion site for hematoma or hemorrhage every 15 minutes for the first hour, every 30 minutes for the subsequent 2 hours, and then hourly until the intravenous catheter is terminated. Evaluate pulses, sensation, color, and temperature of both extremities with each vital sign test. Vital signs and the site are commonly evaluated to find possible complications. (b) Be reminiscent the patient to remain the extremity still and straight. (c) Keep continuous cardiac monitoring during the infusion.	(a) Evaluate important signs, distal pulses, and infusion site regularly as required. (b) Assess response to therapy. (c) Keep bed rest for 6 hours. (d) Assess puncture sites for hemorrhage. (e) Evaluate body fluids, as well as urine, feces, and vomitus for evidence of bleeding. (f) Give platelet-modifying drugs (e.g., aspirin, dipyridamole) as instructed. (g) Report manifestations of reocclusion, as well as changes in the ST segment, chest pain, or dysrhythmias. Early recognition of reocclusion is vital to save myocardial tissue.

**Table 3:** 

Features	Thrombolytics					
	Streptokinase	Urokinase	Anistreplase	Alteplase	Reteplase	Tenecteplase
Plasma half-life (min)	18	15	90–112	4–8	11–14	20
Plasma clearance (mL/min)	10.8 ± 8.8	NR	594 ± 160	572 ± 132	103 ± 138	105
Volume of distribution (L/kg)	5.68	0.04	NR	0.07	NR	NR
Peak plasma level (ng/mL)	NR	2200–2400	NR	1000–4000	4000	>1000
Route of excretion	Renal	Hepatic and renal	NR	Hepatic	Hepatic and renal	Hepatic
Elimination half-life (Alpha phase) (min)	18	NR	70–120	5–10	13–16	11–20
Elimination half-life (Beta phase) (min)	83	NR	NR	72	98–135	41–138
Active metabolite	Unknown	NR	None	None	None	None

^*^NR: not reported.
